# Ca^2+^ Regulates the Kinetics of Synaptic Vesicle Fusion at the Afferent Inner Hair Cell Synapse

**DOI:** 10.3389/fncel.2018.00364

**Published:** 2018-10-17

**Authors:** Chao-Hua Huang, Tobias Moser

**Affiliations:** ^1^Institute for Auditory Neuroscience and InnerEarLab, University Medical Center Göttingen, Göttingen, Germany; ^2^Synaptic Nanophysiology Group, Max Planck Institute for Biophysical Chemistry, Göttingen, Germany; ^3^Collaborative Research Center 889, University of Göttingen, Göttingen, Germany; ^4^Auditory Neuroscience Associated Group, Max Planck Institute for Experimental Medicine, Göttingen, Germany

**Keywords:** ribbon synapse, exocytosis, calcium, cochlea, hearing, quantal hypothesis

## Abstract

The early auditory pathway processes information at high rates and with utmost temporal fidelity. Consequently, the synapses in the auditory pathway are highly specialized to meet the extraordinary requirements on signal transmission. The calyceal synapses in the auditory brainstem feature more than a hundred active zones (AZs) with thousands of releasable synaptic vesicles (SVs). In contrast, the first auditory synapse, the afferent synapse of inner hair cells (IHCs) and type I spiral ganglion neurons (SGNs), typically exhibits a single ribbon-type AZ tethering only tens of SVs resulting in a highly stochastic pattern of transmitter release. Spontaneous excitatory postsynaptic currents (sEPSCs), besides more conventional EPSCs with a single peak, fast rise and decay (compact), also include EPSCs with multiple peaks, variable rise and decay times (non-compact). The strong heterogeneity in size and shape of spontaneous EPSCs has led to the hypothesis of multivesicular release (MVR) that is more (compact) or less (non-compact) synchronized by coordination of release sites. Alternatively, univesicular release (UVR), potentially involving glutamate release through a flickering fusion pore for non-compact EPSCs, has been suggested to underlie IHC exocytosis. Here, we further investigated the mode of release by recording sEPSCs from SGNs of hearing rats while manipulating presynaptic IHC Ca^2+^ influx by changes in extracellular [Ca^2+^] ([Ca^2+^]_e_) and by application of the Ca^2+^ channel antagonist, isradipine, or the Ca^2+^ channel agonist, BayK8644 (BayK). Our data reveal that Ca^2+^ influx manipulation leaves the distributions of sEPSC amplitude and charge largely unchanged. Regardless the type of manipulation, the rate of sEPSC decreased with the reduction in Ca^2+^ influx. The fraction of compact sEPSCs was increased in the presence of BayK, an effect that was abolished when combined with decreased [Ca^2+^]_e_. In conclusion, we propose that UVR is the prevailing mode of exocytosis at cochlear IHCs of hearing rats, whereby the rate of exocytosis and the kinetics of SV fusion are regulated by Ca^2+^ influx.

## Introduction

Established by del Castillo and Katz (1954), the quantal hypothesis of transmitter release has served as a widely accepted model of presynaptic exocytosis. It states that synaptic vesicles (SVs) at the presynaptic active zone (AZ) are released independently of each other, i.e., undergo uniquantal release or univesicular release (UVR), and that neurons primarily regulate the rate at which exocytosis of single SVs occurs. However, exceptions from this scheme exist in neurons and clearly in other secretory preparations. For example, compound and cumulative exocytosis of several vesicles is common in blood cells (Scepek and Lindau, 1993), and compound exocytosis has also been described to govern a fraction of release events at the calyx of Held synapse in the auditory brainstem (He et al., 2009). The resulting exocytosis involves statistical dependent (coordinated) release of several vesicles, i.e., coordinated multivesicular release (MVR). Even with vesicles fusing independently from each other, i.e., an univesicular mode of exocytosis, release evoked by an action potential can involve more than one vesicle, then also said to be multivesicular (Tong and Jahr, 1994). Since this definition is difficult to apply to release at ribbon synapses that is driven by graded potentials rather than action potentials, we resort to the former definition of the mode of release.

**FIGURE 1:**
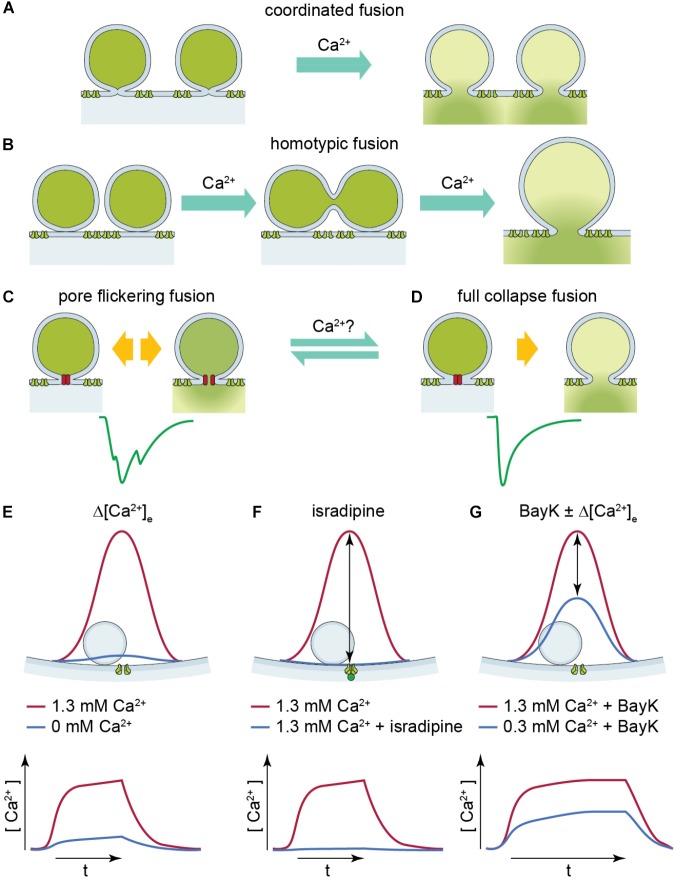
Illustration of two UVR release modes: pore flickering fusion versus full collapse fusion and [Ca^2+^] near SV upon different experimental conditions. **(A,B)** Proposed release mechanisms for multivesicular release: coordinated fusion of multiple vesicles at the same time **(A)** or homotypic fusion of vesicles prior to release **(B)** could lead to large compact sEPSCs. **(C,D)** Proposed release mechanism for univesicular release. The green solid line is a representative non-compact **(C)** or compact **(D)** sEPSC. The hypothetical release mode which leads to non-compact and compact sEPSCs is illustrated above, respectively. Pore-flickering fusion means the SV forms an intermediate fusion pore on the membrane and the pore opens and closes with certain frequencies, and in turn different amount of transmitter is released in different time period. Non-compact sEPSCs reflect such irregular transmitter release. On the other hand, full collapse fusion is the classical SV fusion on to cell membrane. **(E–G)** The red solid line shows [Ca^2+^] level (upper panel) and time course (lower panel) near SV when Ca^2+^ channel opens at 1.3 mM [Ca^2+^]_e_. The blue solid line shows [Ca^2+^] level (upper panel) and time course (lower panel) near SV under **(E)** reduced [Ca^2+^]_e_, **(F)** with isradipine, and **(G)** with BayK in reduced [Ca^2+^]_e_. The [Ca^2+^] upon Ca^2+^ channel opening reaches similar level because the [Ca^2+^]_e_ is the same, so it creates similar driving force; whereas in reduced [Ca^2+^]_e_ with and without BayK [blue solid line in panels **(E,G)**].

The mode of release, UVR or coordinated MVR, is of major relevance for synaptic transmission. The first synapse of the auditory pathway – the afferent synapse of IHCs and spiral ganglion neurons (SGNs)-provides an impressive example (**Figure 1**). Sound encoding at this synapse is thought to rely on a single AZ to drive all spiking in a bipolar postsynaptic neuron (Liberman, 1982). Coordinated MVR of 6 SVs on average (Goutman and Glowatzki, 2007) would require high rates of SV cycling to enable the physiological SGN spike rates of hundreds of Hz. Should UVR prevail, this synapse would uniquely turn a single SV release into a postsynaptic spike, because at least for low rates of transmission almost every EPSC seems to trigger a spike (Rutherford et al., 2012). The hypothesis of coordinated MVR at the afferent hair cell synapse was phrased based on the large, variably sized, and shaped sEPSCs (Glowatzki and Fuchs, 2002; Keen and Hudspeth, 2006). In a series of studies on the afferent synapse of IHCs and SGNs of rats, Glowatzki and Colleagues proposed coordinated MVR in a more (monophasic or compact sEPSCs) or less (multiphasic or non-compact sEPSCs) synchronized manner. They concluded the number of SVs (6) that is on average involved in individual release events is largely independent of voltage and Ca^2+^ at the AZ (Glowatzki and Fuchs, 2002; Goutman and Glowatzki, 2007). In contrast, at the frog auditory hair cell synapse, smaller EPSC amplitudes were observed when the presynaptic hair cell was hyperpolarized to reduce the Ca^2+^ influx by decreasing Ca^2+^ channel open probability (Li et al., 2009). This finding was taken to suggest that MVR occurs and is dependent on voltage-gated Ca^2+^ influx, potentially involving coordination via a common Ca^2+^ nanodomain (Graydon et al., 2011).

A different view was presented by Chapochnikov et al. (2014) in a study on IHC synapses of mice and rats. Based on experiments and mathematical modeling, UVR was suggested as a candidate mode of release at the IHC synapse. This study proposed that non-compact (multiphasic) sEPSCs might arise from release through a flickering fusion pore, while compact sEPSCs were attributed to exocytosis with rapid progression toward full fusion without a noticeable intermediate state (**Figure 1**). Testing potential mechanisms of coordinated MVR, this study did not find larger vesicles at the base of stimulated synapses as would be expected for compound exocytosis and still observed large EPSCs in the absence of extracellular Ca^2+^ (0 mM [Ca^2+^]_e_ with 2 mM EGTA), arguing against the common Ca^2+^ nanodomain hypothesis of MVR. Interestingly, application of the dihydropyridine agonist, BayK, that promotes the bursting opening mode of L-type Ca^2+^ channels, increased the fraction of compact sEPSCs (Chapochnikov et al., 2014). BayK increases the Ca^2+^ channel open probability while the single Ca^2+^ channel current remains unchanged (Hess et al., 1984). Hence the total entry of Ca^2+^ ions rises due to the prolonged Ca^2+^ channel opening time. However, the actual cause of compact sEPSCs being promoted was not identified. Is it the Ca^2+^ channel gating itself directly affecting the fusion machinery (Lerner et al., 2006) or the Ca^2+^ influx that regulates vesicle fusion?

Here, we performed whole-cell patch-clamp recordings from postsynaptic SGN boutons of hearing rats to further elucidate the mode of release at IHC synapses. Pharmacological manipulations of Ca^2+^ influx with isradipine and directly decreasing [Ca^2+^]_e_ lowered sEPSC rate, but did not affect the fraction of compact sEPSCs. The previously described higher prevalence of compact sEPSCs upon BayK (Chapochnikov et al., 2014) was reversed when the single channel current was reduced by lowering [Ca^2+^]_e_ until the whole cell Ca^2+^ current closely matched that prior to BayK. We suggest that longer duration of Ca^2+^ influx at the release site favors full-collapse univesicular fusion.

## Materials and Methods

### Animals and Dissection

P17-19 Wistar rats of either sex were used. Animal handling and experiments complied with national animal care guidelines and were approved by the University of Göttingen Board for animal welfare and the animal welfare office of the state of Lower Saxony. After decapitation, the cochleae were removed from the temporal bones and immersed into the extracellular solution for dissection (in mM): 141.7 NaCl, 5.36 KCl, 10 HEPES, 0.5 MgSO_4_⋅7 H_2_O, 1 MgCl_2_, 0.1 CaCl_2_. 0.5 mg/ml L-glutamine and 2 mg/ml glucose were added just prior to use. The apical turn of cochlea was carefully excised, and placed under a grid in the recording chamber.

### Patch-Clamp Recordings

The postsynaptic boutons of type I SGNs innervating the IHCs were visually identified on a monitor using a 40×/63× water immersion objective lens attached to an upright microscope with differential interference contrast optics (Axioskop FS2, Zeiss), and a 4×/2.5× magnification camera (TILL Photonics). Whole-cell voltage-clamp recordings from postsynaptic boutons of rat type I SGNs of the apical cochlear coil were performed largely as described in previous studies (Glowatzki and Fuchs, 2002; Grant et al., 2011; Rutherford et al., 2012). The recording pipettes, fabricated with a puller (P-87 and P-2000, Sutter) from borosilicate grass with an outer diameter of 1 mm (TW100F-3, World Precise Instruments), had a resistance of 9–11 MΩ after a pressure polishing (Goodman and Lockery, 2000) with a custom-built microforge. The intracellular solution contained (in mM): 137 KCl, 1 Na_2_GTP, 3.5 MgCl_2_, 0.1 CaCl_2_, 5 EGTA, 5 HEPES, and 2.5 Na_2_ATP (pH 7.2 adjusted with KOH, osmolarity ∼290 mOsm). The extracellular solution for recording contained (in mM): 5.8 KCl, 144 NaCl, 0.9 MgCl_2_, 1.3 CaCl_2_, 0.7 NaH_2_PO_4_, 10 D-glucose, and 10 HEPES (pH 7.2 adjusted with NaOH, osmolarity ∼310 mOsm). In the “0 Ca^2+^” experiment, no CaCl_2_ but instead 2 mM of the Ca^2+^ chelator EGTA was added to the extracellular solution (3 mM MgCl_2_ were included to compensate for the omission of Ca^2+^). All chemicals were purchased from Sigma-Aldrich (St. Louis, MO, United States), unless stated otherwise. In all recordings except for the measurement of the resting membrane potential, tetrodotoxin (2.5 μM, Tocris and Santa Cruz) was added to block voltage-gated Na^+^ channels. Five micromolars BayK (Tocris) and 1 μM isradipine (Tocris) were mixed with the extracellular solution prior to perfusion in the experiments of manipulating Ca^2+^ channels. In those experiments, after the control condition, extracellular solution with BayK or isradipine was perfused into the recording chamber through a separate perfusion line. The volume of perfusion line and recording chamber (2 ml) was 5–7 ml. Only those sEPSCs recorded after 10 ml solution flowed in were taken for analysis to ensure the chemical has been applied to the cells. An EPC-10 amplifier controlled by Patchmaster software (both HEKA Electronics, Lambrecht, Germany) was used to sample and filter currents at 20–50 kHz and 5–10 kHz, respectively. sEPSCs were recorded at a holding potential of −90 mV (not corrected for liquid junction potential) at room temperature (21–24°C).

The apparent series resistance (aR_*s*_) was calculated from the height of capacitive transient in response to a 10 mV voltage step. The actual series resistance (R_*s*_) was *post hoc* calculated because the aR_*s*_ is affected by the filtering of amplifier. To obtain the actual amplitude of capacitive transient, we fitted the decay phase of capacitive transient with a double exponential, and extrapolated the fitting curve to time 0. The amplitude of fitting curve at 10 μs was taken for R_*s*_ calculation. The R_*s*_ is roughly 50% smaller than aR_*s*_. Average R_*s*_ values are summarized in **Table 1**. A recording was discarded if *R*_s_ > 100 MΩ or the aRs fluctuates more than 50 MΩ during recording.

**Table 1:** Summary of compact and non-compact sEPSC kinetics in different experimental conditions.

		Control	10 μM Bay K8644	10 μM Bay K8644 in 0.3 mM [Ca^2+^]_e_	Control	0 mM [Ca^2+^]_e_ in 2 mM EGTA transition	Control	1 μM isradipine transition
	*n*	6	6	6	7	6	6	6
	Rs	57.12 ± 4.99	63.33 ± 8.44	71.42 ± 8.04	50.19 ± 3.76	58.85 ± 7.39	36.68 ± 2.32	41.57 ± 7.55
	Frequency (1/s)	7.79 ± 4.13	6.89 ± 1.73	0.93 ± 0.47	11.11 ± 3.95	2.46 ± 1.13	10.56 ± 3.51	0.98 ± 0.21
	Compact EPSC %	52.71 ± 8.01	79.94 ± 8.51	43.57 ± 10.74	59.93 ± 8.57	50.17 ± 13.15	68.05 ± 4.84	60.73 ± 6.48
	Amplitude mean (pA)	417.72 ± 24.86	381.54 ± 19.49	384.47 ± 47.22	377.23 ± 40.76	417.03 ± 55.05	184.89 ± 31.14	214.21 ± 29.41
	Amplitude median (pA)	412.15	398.09	405.34	375.25	387.93	157.28	216.6
Compact EPSC	10–90% rise time (ms)	0.17 ± 0.01	0.14 ± 0.01	0.19 ± 0.01	0.17 ± 0.01	0.16 ± 0.01	0.2 ± 0.02	0.21 ± 0.02
	Decay time (ms)	0.60 ± 0.03	0.61 ± 0.04	0.64 ± 0.04	0.63 ± 0.04	0.68 ± 0.06	0.55 ± 0.05	0.57 ± 0.06
	Charge (pQ)	231.73 ± 13.94	213.92 ± 16.76	230.29 ± 36.13	221.85 ± 23.45	252.65 ± 21.97	97.27 ± 14.15	114.12 ± 12.43
	Amplitude mean (pA)	268.13 ± 35.7	309.31 ± 35.05	225.16 ± 41.27	283.11 ± 35.53	318.89 ± 58.28	107.37 ± 15.53	118.3 ± 15.66
	Amplitude median (pA)	262.00	324.75	236.72	281.04	287.17	99.05	128.1
Non-compact EPSC	Time to peak (ms)	0.81 ± 0.03	0.77 ± 0.04	0.87 ± 0.03	0.79 ± 0.05	0.81 ± 0.03	0.84 ± 0.03	0.86 ± 0.04
	Halfwidth (ms)	1.07 ± 0.07	0.86 ± 0.08	1.17 ± 0.10	1.04 ± 0.1	1.08 ± 0.1	0.89 ± 0.10	1.00 ± 0.1
	Charge (pQ)	245.02 ± 20.87	256.01 ± 23.73	234.49 ± 37.83	254.05 ± 29.36	292.33 ± 37.44	98.91 ± 14.25	112.75 ± 13.66

*Mean ± SEM of parameters describing the waveforms of compact and non-compact EPSCs in all experimental conditions is shown in the table. The kinetics of sEPSCs were not altered significantly in all conditions. Only the frequency and the proportion of compact EPSC changed by manipulating the Ca^2+^ influx at resting IHC potential.*

Spontaneous excitatory postsynaptic currents were detected using a threshold at −15 to −30 pA depending on the noise level. In current clamp experiments on IHC, the same intracellular solution as used for bouton recordings was employed. The intracellular solution for bouton recordings is close to physiological condition which is ideal for measurement of the membrane potential upon the change of extracellular [K^+^]. The membrane potential of IHCs was measured in ruptured-patch clamp configuration, and the resting membrane potential read out when no current was applied.

### Statistical Analysis

For detection and analysis of EPSCs, Mini Analysis software (Synaptosoft) was used with a detection threshold set at 3–5 times greater than the root mean square (rms) of the baseline noise. To classify EPSCs into compact or non-compact EPSCs, the method described by (Chapochnikov et al., 2014) was employed. The data were further analyzed using *Excel* and *Igor Pro 6* (WaveMetrics) and prepared for presentation using *Adobe Illustrator*. Averages were expressed as mean ± standard error of the mean (SEM). In order to compare two samples, data sets were tested for normal distribution (Jarque–Bera test) and equality of variances (*F*-test) followed by two-tailed paired Student’s *t*-test, or, when data were not normally distributed and/or variance was unequal between samples, the paired two-tailed Mann–Whitney-Wilcoxon test was used.

## Results

Here we studied the mode of SV release (**Figure 1**) and its regulation by Ca^2+^ at the rat IHC synapse after hearing onset by performing whole-cell patch-clamp recordings of sEPSCs from the postsynaptic SGNs at postnatal days (P) P17–P19, while manipulating Ca^2+^ influx in the unclamped IHCs. In some recordings, where the release frequency was too low to collect enough events for sufficient statistics, 10–15 mM [K^+^]_e_ was applied to depolarize the IHC by 10–20 mV (**Supplementary Figures S1B,C**). Under the basal conditions (5.8 mM [K^+^]_e_, 137 mM [K^+^]_i_) the membrane potential of IHCs measured in current-clamp was stable and more hyperpolarized (∼−74 mV) than that thought to occur *in vivo* (∼−55 mV when the mechanotransducer current is not as much inhibited by extracellular Ca^2+^, Johnson et al., 2011). Therefore, we consider the depolarization of IHCs induced by 10–15 mM [K^+^]_e_ to be closer to the physiological resting membrane potential and hence, we generally refer to these EPSC as sEPSCs.

### sEPSC Amplitude and Charge Were Not Altered Upon Diminishing Ca^2+^ Influx

To evaluate if the observed sEPSCs could be further broken down to smaller units (miniature EPSC, mEPSCs), we reduced the presynaptic Ca^2+^ influx by (i) diminishing [Ca^2+^]_e_ to 0 mM or (ii) reducing the Ca^2+^ channel opening probability with the dihydropyridine Ca^2+^ channel antagonist, isradipine.

**FIGURE 2:**
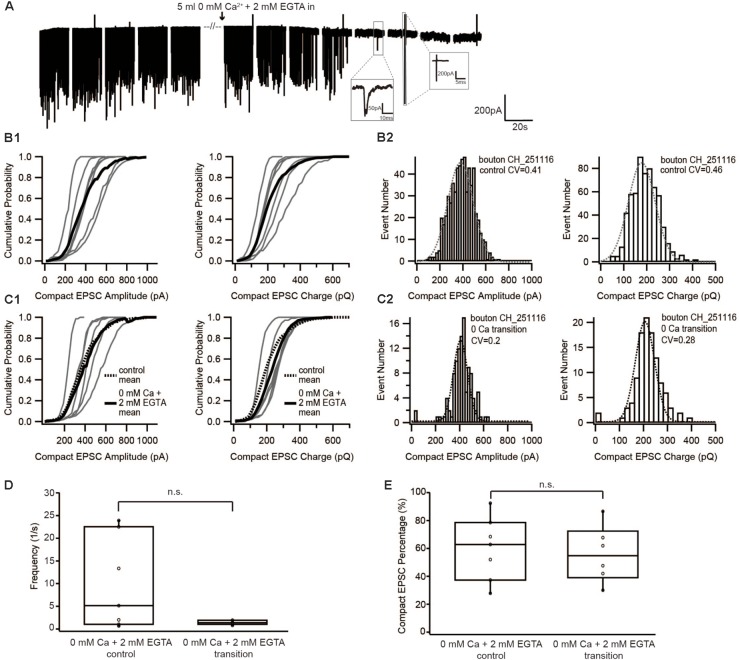
Diminishing Ca^2+^ influx by reducing extracellular [Ca^2+^] neither changed the size of EPSC nor the proportion of compact EPSCs. **(A)** Example traces of recordings before, during and after the perfusion of 0 mM Ca^2+^ + 2 mM EGTA. The frequency suddenly dropped to zero when the [Ca^2+^]_e_ was completely removed. The insets show an example of a real event and of noise: a real sEPSC has slower time constant while a noise has rather fast time constant and an up-and-down signal. **(B1,C1)** Cumulative probability function of the compact EPSC amplitude (left) and charge (right) before **(B1)** and during perfusion of 0 mM [Ca^2+^]_e_ + 2 mM EGTA **(C1)**. The bold solid line is the mean of all recordings in each experiment group. For comparison with the 0 mM Ca^2+^ condition, the mean of the control condition is shown on panel **(C1)** as bold dashed line. **(B2,C2)** Histogram of compact EPSC amplitude (left) and charge (right) from a representative bouton before **(B2)** and in the transition time while perfusing 0 mM [Ca^2+^]_e_ + 2 mM EGTA **(C2)**. The dashed line is a Gaussian function fitted to the data. **(D)** Box-Whisker plot of EPSC frequency in different experimental conditions (*n* = 7 in control condition; *n* = 6 in 0 mM Ca^2+^+ 2 mM EGTA). There was no statistically significant difference between the two groups (Wilcoxon Rank test, *p* = 0.16). **(E)** Box-Whisker plot of compact EPSC percentage in different experimental conditions. There is no significant difference between the control condition and the reduced Ca^2+^ influx condition (*p* = 0.67). Box plots show 10, 25, 50, 75, and 90th percentiles with the individual data points overlaid.

In the first set of experiments, the extracellular solution containing 1.3 mM [Ca^2+^] was slowly replaced by nominally Ca^2+^-free extracellular solution with 2 mM EGTA added. Instead of starting the recording after the extracellular Ca^2+^ was completely removed for at least 5 min as done in Chapochnikov et al. (2014), sEPSCs were recorded during the entire period of Ca^2+^ washout. Due to the near complete absence of release upon total Ca^2+^ deprivation, analysis was performed on sEPSCs recorded during the perfusion of the Ca^2+^-free solution after the frequency had substantially dropped and before the sEPSCs completely stopped (**Figures 2A,D**).

If compact sEPSCs were caused by MVR synchronized by Ca^2+^, the percentage of smaller compact sEPSCs and/or non-compact sEPSCs would increase as a consequence of the reduced SV’s release probability due to low Ca^2+^ influx. Therefore, we first studied the prevalence of compact sEPSCs; however, their proportion remained unchanged (**Figure 2E**). We then analyzed the amplitude and charge of compact (monophasic) sEPSCs under the two conditions. We focused on compact sEPSCs given that the amplitude of non-compact sEPSCs may be affected by factors other than the amount of the neurotransmitter content of the SV (Chapochnikov et al., 2014). The amplitude distribution of compact sEPSC had small coefficient of variance (CV, 0.3–0.4) and could be fitted with a single Gaussian, both, before and during perfusion (**Figure 2B2**: control, **Figure 2C2**: 0 Ca^2+^). The amplitude and charge of compact sEPSCs remained unchanged after perfusion of Ca^2+^-free saline (0 mM [Ca^2+^] + 2 mM EGTA, **Figures 2B,C**). The same observation was true for non-compact sEPSCs (**Table 1**). Importantly, there was no obvious appearance of additional small sEPSCs when Ca^2+^ influx was reduced. Taken together, our data argue against Ca^2+^-synchronized MVR hypothesis at near IHC resting potential.

**FIGURE 3:**
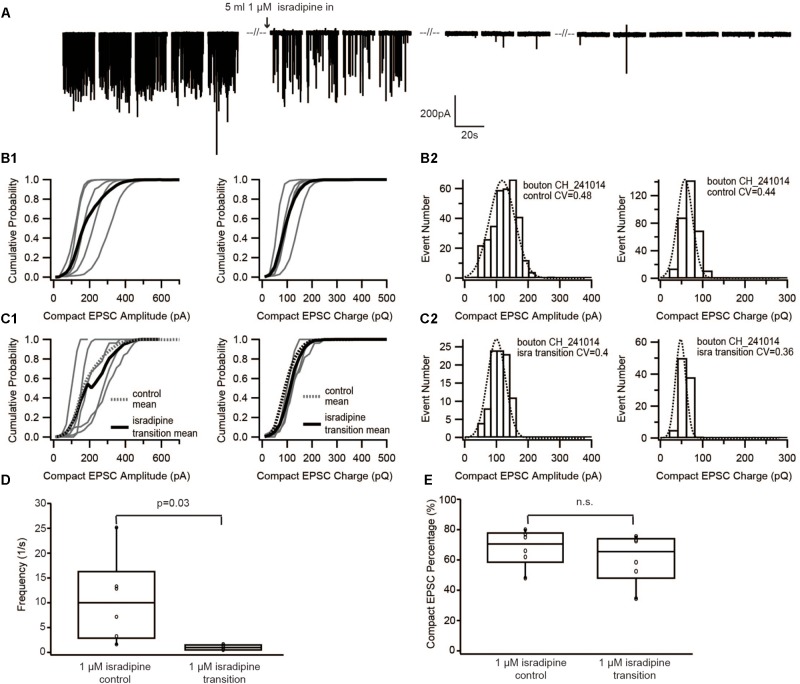
Diminishing Ca^2+^ influx by blocking Ca^2+^ channels with isradipine neither changed the size of EPSC nor the proportion of compact EPSCs. **(A)** Example traces of recordings before/during/after perfusion with 1 μM isradipine. **(B1,C1)** Cumulative probability function of the compact EPSC amplitude (left) and charge (right) before perfusion of isradipine **(B1)**, and during perfusion of 1 μM isradipine **(C1)**. The bold solid line is the mean of all recordings in each experimental condition. For comparison with the isradipine condition, the mean of control group condition was shown on panel **(C1)** as bold dash line. **(B2,C2)** Histogram of compact EPSC amplitude (left) and charge (right) from a representative bouton in the control condition **(B2)**, and while perfusing 1 μM isradipine **(C2)**. **(D)** Box-Whisker plot of EPSC frequency in different experimental conditions. The frequency of sEPSCs dropped down significantly (paired Wilcoxon signed test: *p* = 0.03, *n* = 6 for each group). **(E)** Box-Whisker plot of compact EPSC percentage in different experimental conditions. There is no significant difference between the control group and its reduced Ca^2+^ influx group (paired Wilcoxon signed test: *p* = 0.22, *n* = 6 in each group). Box plots show 10, 25, 50, 75, and 90th percentiles with the individual data points overlaid.

To further corroborate this result, we used a different approach to decrease the Ca^2+^ influx of the presynaptic IHC. For this purpose, we lowered Ca^2+^ channel opening probability by slowly perfusing 1 μM isradipine (Koschak, 2001; Brandt et al., 2003). Similar to the previous experiment, as soon as isradipine solution completely replaced the control bath solution, no event was observed for at least 1 min of recordings (**Figure 3A**). Therefore, the analysis was performed on sEPSCs recorded during the isradipine-perfusion, after we observed a substantial reduction of sEPSC-frequency (**Figure 3D**). The proportion of compact sEPSCs remained unaffected (*p* = 0.22, **Figure 3E**). The sEPSC amplitude distribution showed a small CV (0.3–0.4) and could be fitted with a single Gaussian in both before and during isradipine perfusion (**Figures 3B2,C2**). Moreover, the charge of compact sEPSCs (**Figures 3B2,C2**) and non-compact sEPSCs was comparable to control conditions (**Table 1**). In summary, we consider this invariance of EPSC amplitude despite a massive reduction of release probability to indicate UVR as the prevailing release mechanism.

### Total Ca^2+^ Entry Rather Than the Size of Ca^2+^ Current Seems to Determine the Share of Compact and Non-compact sEPSCs

**FIGURE 4:**
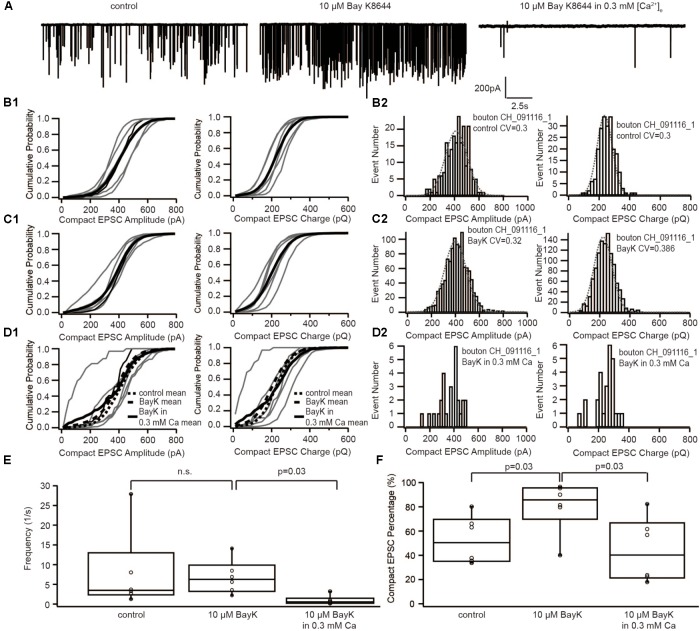
The dihydropyridine Ca^2+^ channel agonist, BayK8644, increased the occurrence of compact sEPSC, which could be reversed by reducing [Ca^2+^]_e_. **(A)** Example traces of recordings in control, with 10 μM BayK8644 and with 10 μM BayK8644 in 0.3 mM [Ca^2+^]_e_. **(B1–D1)** Cumulative probability function of the compact sEPSC amplitude (left) and charge (right) in control group **(B1)**, in 10 μM BayK8644 **(C1)**, and in 10 μM BayK8644 with 0.3 mM [Ca^2+^]_e_ **(D1)**. The bold solid line is the mean of all recordings in each experiment group. For comparison, the mean curve of control, 10 μM BayK8644 are shown in panel **(D1)** as bold dash lines. **(B2–D2)** Histogram of compact sEPSC amplitude (left) and charge (right) from a representative bouton in control group **(B2)**, in 10 μM BayK8644 **(C1)**, and in 10 μM BayK8644 with 0.3 mM [Ca^2+^]_e_ **(D1)**. **(E)** Box-Whisker plot of sEPSC frequency in different conditions. There is no significant difference between control and 10 μM BayK8644 (*n* = 6, *p* = 0.43 with paired Wilcoxon signed test), but the frequency in 10 μM BayK8644 with 0.3 mM [Ca^2+^]_e_ was reduced significantly (*n* = 6, *p* = 0.03 with paired Wilcoxon signed test). **(F)** Box-Whisker plot of compact sEPSC percentage in different conditions. Ten micromolars BayK8644 increased the proportion of compact sEPSC significantly (*n* = 6, *p* = 0.03 with paired Wilcoxon signed test). When the [Ca^2+^]_e_ was reduced to 0.3 mM, the compact sEPSC percentage decreased significantly (*n* = 6, *p* = 0.03 with paired Wilcoxon signed test). Box plots show 10, 25, 50, 75, and 90th percentiles with the individual data points overlaid.

If sEPSCs recorded in the postsynaptic bouton of SGNs indeed reflect UVR at IHC AZs, compact and non-compact EPSCs likely represent two kinetic variants of SV fusion, for example, full-collapse fusion and fusion preceded by fusion pore flickering. To decipher the mechanism regulating the kinetics of SV fusion, we recorded sEPSCs under three subsequent conditions: (i) control, (ii) with 5 μM BayK, and (iii) with 5 μM BayK + 0.3 mM [Ca^2+^]_e_ (**Figure 4A**). As reported previously (Chapochnikov et al., 2014), the sEPSC frequency remained on average the same in BayK as in control, yet it declined significantly (*p* = 0.03, *n* = 6) when lowering [Ca^2+^]_e_ to 0.3 mM in the continued presence of BayK (**Figure 4E**). At 0.3 mM [Ca^2+^]_e_, Ca^2+^ influx of IHCs at −50 mV was close to the one observed during the control before the perfusion with BayK (**Supplementary Figure S1A**). Therefore, this manipulation removed the effect of higher Ca^2+^ influx and allowed us to isolate the impact of the increased open probability. The amplitude and charge of compact sEPSCs were not affected regardless of the [Ca^2+^]_e_ (**Figures 4B–D**). This suggests that sEPSCs in the presence of BayK and without depolarization of IHCs result from UVR. Importantly, the proportion of compact sEPSCs was increased with BayK, but this increase was reversed when lowering [Ca^2+^]_e_ (**Figure 4F**). The results suggest that the shift from non-compact to compact sEPSCs requires an increase of the total Ca^2+^ entry (incoming Ca^2+^ charge) at the release sites.

## Discussion

The mode of SV release is a key property of synaptic transmission. Which release mode – coordinated MVR or UVR – prevails at the rat inner hair cell (IHC) ribbon synapse has remained an open question. Here we studied the effects of manipulating Ca^2+^ influx on the size and shape of sEPSCs at the IHC ribbon synapse of rats after the onset of hearing. Our recordings were done from rat SGNs that encode information about high frequency sound information, such that caution applies when generalizing conclusions to synapses signaling information about low sound frequencies. Reducing Ca^2+^ influx and thereby release probability by decreasing the single channel current or the number of open channels did not change the size and shape of sEPSCs, which argues in favor of UVR prevailing at the IHC ribbon synapse. Prolonging channel open time increased the proportion of compact (monophasic) sEPSCs, which could be reversed when simultaneously lowering the single channel current. In the framework of the UVR hypothesis this might indicate that higher time-averaged [Ca^2+^] at the release site favors rapid transition to irreversible fusion pore opening.

### What Is the Basic Unit of Neurotransmitter Release at the IHC Synapse?

Recordings of postsynaptic currents from the IHC synapse reveal a striking heterogeneity in amplitude and shape of sEPSCs, which seemed at odds with the quantal hypothesis of neurotransmission by Katz and Miledi (1967). Instead, the hypothesis of coordinated MVR has been put forward by Glowatzki and Fuchs (2002). Their notion that an average of six SVs fuse in a coordinated manner would also explain the large sEPSC size (hundreds of Pico amperes). A study of fluctuations in presynaptic exocytic membrane capacitance changes in IHCs from mice after hearing onset supported the occurrence of fusion events with quantal size larger than a single SV in 35% of the cases (Neef et al., 2007). They suggested that coordinated MVR occurs, but involves fewer SVs than previously proposed. MVR was further supported by work on other hair cell synapses (Keen and Hudspeth, 2006; Li et al., 2009). Moreover, parallel research in the retina also indicated the presence of coordinated MVR at the ribbon synapses of retinal bipolar cells (Singer et al., 2004; Matthews and Sterling, 2008; Bedggood and Metha, 2013). Starting with an attempt to decipher the mechanism(s) underlying coordinated MVR at the IHC synapse by experiments and computational modeling, Chapochnikov et al. (2014) arrived at the idea that UVR through a dynamic fusion pore might be an alternative explanation for the heterogeneity of amplitude and shape of sEPSCs at the IHC synapse.

The rationale of the current study followed the general principle to characterize the minimal quantal size of a synapse by reducing release probability to elicit the minimum output causing the mEPSC. Assuming that the amount of neurotransmitter per SV follows a normal distribution and the postsynaptic receptors are not saturated (the relationship of the amount of neurotransmitter and EPSC amplitude is linear), the mEPSC amplitude distribution should be Gaussian with a small coefficient of variation (CV, of ∼0.4, Neef et al., 2007). The amplitude and charge distributions of the compact sEPSCs, recorded in the present study could be approximated by Gaussian functions and had CVs in the range of 0.3–0.4, as if they were mEPSCs. Lowering the release probability through Ca^2+^ influx reduction, did not change the amplitude and charge distributions of compact sEPSCs (**Figures 2**, **3** and **Table 1**), indicating that compact sEPSCs most likely reflect release from single SVs and argues that UVR prevails at the IHC synapse of hearing rats. Since the charge of compact and non-compact sEPSCs were not significantly different (**Table 1**), we suggest that non-compact sEPSCs, also reflect UVR likely involving release through a dynamic fusion pore.

In contrast, at frog hair cell synapses it was shown that lower Ca^2+^ influx revealed a smaller EPSC amplitude indicating that there, not all sEPSCs were mEPSCs (Li et al., 2009). This could be accounted by the anatomical structure difference between rat and frog hair cell synapses. The postsynaptic bouton of frog hair cell is a calyx-like structure, which receives SV release from multiple AZs whereas the bouton of rat hair cell has a one-to-one relationship to the presynaptic AZ as it receives only SV release from one AZ. This might explain why, at the frog hair cell synapse, the sEPSC involves release of more than one SV.

### Are Compact and Non-compact sEPSCs Kinetically Different Schemes of SV Fusion?

The finding of non-compact (multiphasic) sEPSCs was influential for phrasing the concept of MVR at the IHC synapse and therein they were interpreted as less synchronized fusion of SVs (Glowatzki and Fuchs, 2002). The abundance of non-compact sEPSCs lowers as the IHC synapse matures, yet can still be observed even at 2 months of age in rats, and were thought to be less efficient in triggering action potential when compared to compact sEPSCs (Grant et al., 2010). The similarity in the charge of the compact and non-compact sEPSCs was taken to argue for UVR to prevail at the IHC synapse (Chapochnikov et al., 2014). Within the framework of UVR the non-compact sEPSCs were thought to reflect protracted release through a flickering fusion pore. From these previous observations and the present findings, we favor the hypothesis that compact and non-compact sEPSCs reflect kinetically different schemes of univesicular fusion.

The canonical scheme of full-collapse fusion of SVs onto the cell membrane and the neurotransmitter being released completely at once has also been favored for retinal ribbon synapses (Llobet et al., 2003; Zenisek et al., 2003). We favor the interpretation that rapid progression to full-collapse fusion underlies compact sEPSCs. Other than that, incomplete vesicle fusion, kiss-and-run or pore-flickering, has been reported in retinal ribbon synapses (Grabner and Zenisek, 2013), conventional synapses (Staal et al., 2004; He et al., 2009; Zhang et al., 2009) and chromaffin cells/PC12 cells (Chow et al., 1992; Albillos et al., 1997; Mellander et al., 2012). Many factors such as lipid composition of the plasma membrane (Amatore et al., 2006; Uchiyama et al., 2007; Mellander et al., 2012), temperature (Pihel et al., 1996; Haynes et al., 2007), and osmolarity (Borges et al., 1997) have been suggested to regulate the incomplete fusion pore. However, the molecular mechanism is not yet fully understood.

Interestingly, at the IHC ribbon synapse, the fraction of compact sEPSCs was increased by two opposing manipulations of Ca^2+^ influx: abolishing Ca^2+^ influx at IHC synapses by omitting extracellular Ca^2+^ in mice and increasing Ca^2+^ influx by application of BayK in rats (Chapochnikov et al., 2014). In the present study, recording from rat IHC synapses, we could not reproduce the elevation of the share of compact sEPSCs during the wash-out of Ca^2+^ from the bath solution. The discrepancy might relate to differences in methods (recording while reducing [Ca^2+^]_e_ here vs. steady state following wash-out of Ca^2+^, Chapochnikov et al., 2014) and in biology (rat IHC synapses here after vs. mouse IHC synapses before hearing onset there). In keeping with our previous finding, the fraction of compact sEPSCs increased in the presence of BayK (**Figure 4**). However, this effect was reversed upon subsequently decreasing [Ca^2+^]_e_ to 0.3 mM in the continued presence of BayK, which in separate experiments was shown to closely match the IHC Ca^2+^ influx to the pre-BayK level. A potential explanation within the framework of the UVR hypothesis is that the increased Ca_V_1.3 channel open probability with BayK favors rapid transition to irreversible fusion pore opening due to higher time-averaged [Ca^2+^] at the release site. This interpretation, however, contrasts previous findings in chromaffin cells, where higher Ca^2+^ supported longer fusion pore openings (Zhou et al., 1996).

The alternative interpretation that the gating mode of Ca_V_1.3 channel affects the fusion process by direct protein-protein interaction (Lerner et al., 2006) seems less likely given the dependence of the phenomenon on single channel current. Hence, we favor the hypothesis of a complex regulation of the fusion process by Ca^2+^: while the instant increase of [Ca^2+^] at the release site upon the opening of Ca^2+^ channels initiates the fusion process, the duration of the [Ca^2+^] elevation (channel opening) might determines the mode of fusion process. A longer duration of Ca^2+^ elevation at the release site would promote full-collapse fusion. Further experiments, such as paired recordings of IHC and post-synaptic bouton and cell-attached membrane capacitance measurements from IHCs, and computational modeling are required to further study the mechanism of exocytosis at the IHC synapse.

## Data Availability

The raw data supporting the conclusions of this manuscript will be made available by the authors, without undue reservation, to any qualified researcher.

## Author Contributions

C-HH and TM designed the study. C-HH performed the patch-clamp measurements and analysis. C-HH and TM prepared the manuscript.

## Conflict of Interest Statement

The authors declare that the research was conducted in the absence of any commercial or financial relationships that could be construed as a potential conflict of interest.

## Supplementary Material

FIGURE S1 **(A)** The I-V curve of Ca^2+^ influx at IHCs recorded at whole cell patch clamp configuration (before liquid junction potential correction; liquid junction potential calculated with Igor built-in macro was 4.3 mV). The black open circle is control group (*n* = 6); black solid circle is with 10 μM BayK (*n* = 6), and gray open circle is with 10 μM BayK in 0.3 mM [Ca2+]e (*n* = 6). The Ca^2+^ influx at -55 mV with BayK in 0.3 mM [Ca^2+^]_e_ is close to that of control whereas the Ca^2+^ influx with BayK at -55 mV is more than 2 times larger. **(B)** A representative trace of membrane potential measurement of IHCs during whole cell current clamp. The resting membrane potential was around -70 mV (when the current was clamped to 0). When solution containing additional 10 mM K^+^ was perfused into the recording chamber, IHCs were depolarized to ~-50 mV which is close to the resting membrane potential of IHCs in physiological condition. **(C)** Box-Whisker plot of IHC membrane potential before, during and after perfusion of 10 mM K^+^. The open circle is the average of those data points in one IHC in the time window indicated by the black open rectangle in B (*n* = 5). Box plots show 10, 25, 50, 75 and 90th percentiles with the individual data points overlaid.
