# Supplementary figures and images for: Ca2+ Regulates the Kinetics of Synaptic Vesicle Fusion at the Afferent Inner Hair Cell Synapse
Source: Front Cell Neurosci. 2018 Oct 17;12:364. doi: 10.3389/fncel.2018.00364 (PMC6199957; doi:10.3389/fncel.2018.00364)

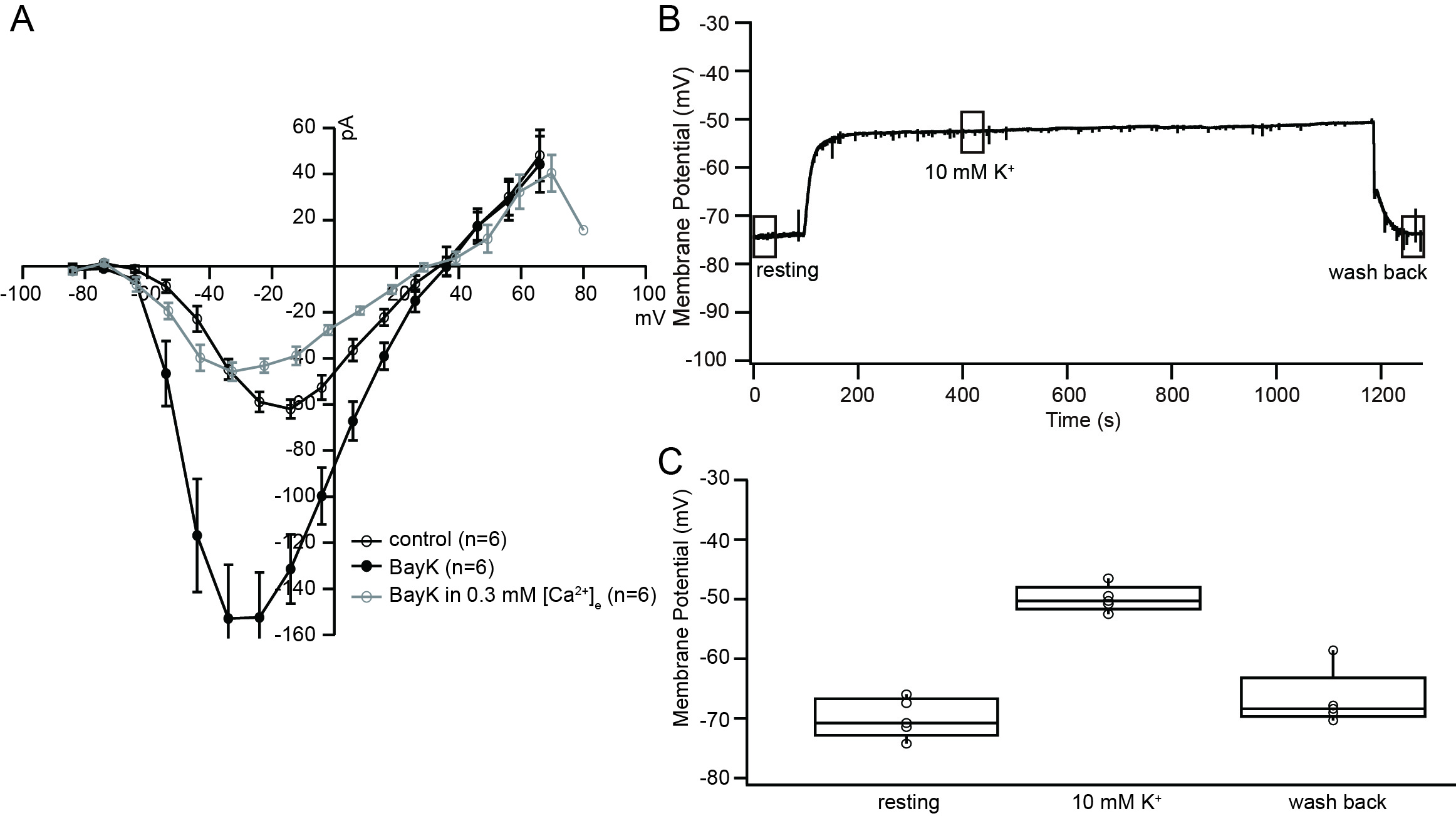

Supplement: FIGURE S1 — (A) The I-V curve of Ca2+ influx at IHCs recorded at whole cell patch clamp configuration (before liquid junction potential correction; liquid junction potential calculated with Igor built-in macro was 4.3 mV). The black open circle is control group (n = 6); black solid circle is with 10 μM BayK (n = 6), and gray open circle is with 10 μM BayK in 0.3 mM [Ca2+]e (n = 6). The Ca2+ influx at -55 mV with BayK in 0.3 mM [Ca2+]e is close to that of control whereas the Ca2+ influx with BayK at -55 mV is more than 2 times larger. (B) A representative trace of membrane potential measurement of IHCs during whole cell current clamp. The resting membrane potential was around -70 mV (when the current was clamped to 0). When solution containing additional 10 mM K+ was perfused into the recording chamber, IHCs were depolarized to ~-50 mV which is close to the resting membrane potential of IHCs in physiological condition. (C) Box-Whisker plot of IHC membrane potential before, during and after perfusion of 10 mM K+. The open circle is the average of those data points in one IHC in the time window indicated by the black open rectangle in B (n = 5). Box plots show 10, 25, 50, 75 and 90th percentiles with the individual data points overlaid. [file Image_1.jpg]
